# Whole-Genome Sequencing and Bioinformatic Analysis of Environmental, Agricultural, and Human *Campylobacter jejuni* Isolates From East Tennessee

**DOI:** 10.3389/fmicb.2020.571064

**Published:** 2020-11-05

**Authors:** Brittni R. Kelley, J. Christopher Ellis, Annabel Large, Liesel G. Schneider, Daniel Jacobson, Jeremiah G. Johnson

**Affiliations:** ^1^Department of Microbiology, The University of Tennessee, Knoxville, Knoxville, TN, United States; ^2^Biosciences, Oak Ridge National Laboratory, Oak Ridge, TN, United States; ^3^Department of Animal Science, The University of Tennessee, Knoxville, Knoxville, TN, United States; ^4^Bredesen Center, The University of Tennessee, Knoxville, Knoxville, TN, United States

**Keywords:** *Campylobacter* (*C. jejuni*), whole-genome sequencing (WGS), environmental isolation, agricultural isolates, human campylobacteriosis

## Abstract

As a leading cause of bacterial-derived gastroenteritis worldwide, *Campylobacter jejuni* has a significant impact on human health in both the developed and developing worlds. Despite its prevalence as a human pathogen, the source of these infections remains poorly understood due to the mutation frequency of the organism and past limitations of whole genome analysis. Recent advances in both whole genome sequencing and computational methods have allowed for the high-resolution analysis of intraspecies diversity, leading multiple groups to postulate that these approaches may be used to identify the sources of *Campylobacter jejuni* infection. To address this hypothesis, our group conducted a regionally and temporally restricted sampling of agricultural and environmental *Campylobacter* sources and compared isolated *C. jejuni* genomes to those that caused human infections in the same region during the same time period. Through a network analysis comparing genomes from various sources, we found that human *C. jejuni* isolates clustered with those isolated from cattle and chickens, indicating these as potential sources of human infection in the region.

## Introduction

As a leading cause of bacterial-derived gastroenteritis worldwide, *Campylobacter* species have a significant impact on human health (Kaakoush et al., 2015; Crofts et al., 2018) with approximately 96 million global cases (Kirk et al., 2015) and 1.3 million cases in the United States, annually (Fitzgerald et al., 2016). Symptoms of acute campylobacteriosis in the developed world typically include bloody and/or watery diarrhea, lethargy, and abdominal cramps (Connerton and Connerton, 2017). While the majority of cases are self-limiting and subside after several days, several post-infectious disorders have been associated with *Campylobacter* infections, including the development of Guillain-Barré Syndrome, post-infectious reactive arthritis, and irritable bowel syndrome (Backert et al., 2017; Halpin et al., 2018). In the developed world, consumption of undercooked or improperly prepared poultry has historically been implicated as the predominant source of human infection (Young et al., 2007; Kaakoush et al., 2015); however, direct contact with live cattle, pigs, sheep, and contaminated drinking water may also serve as sources of infection (Ashbolt, 2004; Mou et al., 2015; Kempf et al., 2017; Sacher et al., 2018). Because *Campylobacter* infections significantly impact human health and there are several potential sources of human infection, it is important to public health that these sources and the proportion of human infections attributed to each be thoroughly understood in both the developed and developing worlds.

Relative to foodborne pathogens like *E. coli* and *Salmonella*, successful source-tracking of *Campylobacter* has proven challenging. For example, pulse-field gel electrophoresis (PFGE) was highly impactful in identifying foodborne outbreaks and performing bacterial source-tracking of *E. coli* and *Salmonella* serotypes (Johnson et al., 1995; Fugett et al., 2007). Unfortunately, since this method relies on gel-based resolution of genomic restriction fragments, it can fail to discriminate between strains of bacterial species that experience even minor genomic variation, like *Campylobacter*, since the restriction fragment pattern can be altered (Fitzgerald et al., 2005). To circumvent these limitations, multi-locus sequence typing (MLST) was used by state and federal public health agencies to discriminate between strains and identify sources of infections for pathogens that were not amenable to PFGE. MLST differs from PFGE in that seven conserved housekeeping genes are amplified and sequenced, allowing for a SNP-based comparison of sequences to those previously deposited in online databases. This analysis allowed a queried strain to be designated as a sequence type (ST), which allowed for the assignment of particular STs to specific sources (Kittl et al., 2013; Kovanen et al., 2014). Similar to PFGE, this method works well to discriminate between strains of genomically stable pathogens, but has limited efficacy when distinguishing between strains of *Campylobacter* due to inherent hypervariability of the genome (Parkhill et al., 2000). Such variability has led to an overabundance of ST assignments within *Campylobacter*, making outbreak detection and source attribution challenging (Thépault et al., 2018b). Recently, U.S. public health agencies have begun shifting from PFGE- and MLST-based analyses to whole genome sequencing (WGS) approaches. These changes were instituted due to several perceived advantages, including the ability to analyze bacterial genomes with single nucleotide-level resolution, regular access of more laboratories to sequencing technology, rapidly decreasing costs of sequencing a genome, and increased speed of sequencing (Salipante et al., 2015).

Using these WGS technologies, groups have begun investigating the genetic relatedness of *Campylobacter* isolates from various sources (Dearlove et al., 2016; Kovanen et al., 2019), including whole genome MLST (wgMLST) analysis (Cody et al., 2013). For example, wgMLST has been used to examine *C. jejuni* and *C. coli* clonal complexes (Sheppard et al., 2012), the presence of *Campylobacter* antibiotic resistant genotypes (Zhao et al., 2016), and the comparison of isolates recovered in processing plants and on chicken meat (Ma et al., 2014; Guyard-Nicodème et al., 2015). Additionally, wgMLST studies have analyzed isolates from agricultural sources, including cattle and chickens, to examine for links to human infections based on genomic similarity and ST assignments (Sheppard et al., 2009; Oporto et al., 2011; Sheppard et al., 2012; Rosner et al., 2017). Due to the challenges of identifying *Campylobacter* outbreaks in real time, clinical cases were considered to occur sporadically. However, retrospective studies using WGS in conjunction with epidemiological data have been employed to investigate the genetic relatedness of clinical isolates and identify potential sources indicated by epidemiological data (Revez et al., 2014a, b; Clark et al., 2016; Moffatt et al., 2016; Joensen et al., 2018; Montgomery et al., 2018; Oakeson et al., 2018).

Despite the success of these studies and the increasing availability of sequencing technologies in health department laboratories, the ability to process and analyze the resulting sequences in a timely and accurate manner remains a limiting factor in public health investigations (Fricke and Rasko, 2014). Reliable protocols to perform source-tracking have not been verified for most pathogens, including *Campylobacter* (Dearlove et al., 2016), and the lack of continued systemic surveillance and reliance on sequence repositories can make the real-time detection of outbreaks difficult (Llarena et al., 2017). Taken together, these observations indicate that it is becoming increasingly urgent that streamlined surveillance and WGS-based approaches be developed and vetted for public health investigations, especially for regions with limited resources.

The objective of this work was to collect agricultural, environmental, and clinical *C. jejuni* isolates from East Tennessee during a defined time period and with collaborators at Oak Ridge National Laboratory (ORNL) conduct a novel network-based analysis to identify potential sources of human infections in the region. A total of 630 samples were collected between October 2016 and October 2018, resulting in 144 PCR-confirmed *C. jejuni* isolates. Subsequent whole-genome sequencing and assembly resulted in 80 high quality genomes collected during this study, while an additional 87 high quality genome assemblies were identified from the GenomeTrakr database. Together, these 167 genomes were incorporated into a robust reference-independent network analysis. Using this bioinformatic approach, we found that the human isolates clustered with those from cattle and chickens, which are known to be common sources of human *Campylobacter* infections. Working within the temporal and geographical constraints of this study, we were able to isolate C. *jejuni* from a variety of sources highlighting the importance of broad surveillance, while supporting the potential of whole-genome sequencing for source-tracking by utilizing a novel network analysis approach for comparison of isolates from different sources.

## Materials and Methods

This study was an observational survey that utilized samples from confirmed human cases and convenience sampling from water, foods, and fresh excreted feces from domestic animals over the course of 2 years.

### Regional Sampling of Water and Food

Samples from local fresh water sources (rivers, streams, and tributaries) in East Tennessee were aseptically collected in sterile 100 mL screw top glass bottles. A minimum of two samples were taken from each sampling site. Samples were stored on ice until filtration with a 0.2 μm vacuum filter. Filters were aseptically removed and placed in a 15 mL tube with 5 mL sterile 1x PBS and vortexed vigorously for 20 s before serial dilution and plating on *Campylobacter-*selective media consisting of Mueller-Hinton (MH) agar supplemented with 10% defibrinated sheep blood, cefoperazone (40 μg/ml), cycloheximide (100 μg/ml), trimethoprim (10 μg/ml), and vancomycin (100 μg/ml). Plates were incubated for 48 h under microaerobic conditions (85% N_2_, 10% CO_2_, 5% O_2_) at 37°C.

Meat (raw chicken, pork, and beef), fruit (unwashed berries, citrus fruit, apples, bananas, peaches, and plums), and vegetable (unwashed potatoes, kale leaves, and carrots) samples were obtained from local grocery chains and farmers markets in Knox County, TN and the surrounding region of eastern Tennessee. Food samples were cut into approximately 3 cm^3^ pieces using a disinfected cutting board and razor blade. The resulting cubes were placed in sterile 15 ml conical tubes with enough MH broth to cover the samples (approximately 3 mls) and allowed to shake overnight under microaerobic conditions at 37°C. Following incubation, samples were serially diluted and plated on *Campylobacter-*selective media as previously described. Only city and sample type were collected as metadata.

### Regional Sampling of Animal Feces

Convenience fecal samples obtained from animals under observation for routine veterinary care from the University of Tennessee College of Veterinary Medicine were collected from the outside of examination gloves and weighed out in 200 mg aliquots and placed in sterile 15 ml conical tubes containing 2 mls sterile 1x PBS. Samples were then vortexed vigorously for 20 s before serial dilution and plating as previously described for other samples. Organically raised chicken fecal isolates were obtained from fecal samples on the ground using sterilized tongue depressors and sterile 15 ml conical tubes, before processing and plating for isolation as described above. Only city and host species were collected as metadata.

### Obtaining Regional Human Isolates

*Campylobacter jejuni* strains used in this study were isolated previously from human clinical samples by the Tennessee Department of Health using commercially available *Campylobacter* blood free selective media (CCDA) plates. After a 48 h incubation at 42°C in a GasPak container with a Campy sachet, isolates were used to conduct a Gram stain, hippurate hydrolysis assay, catalase, indole, and oxidase test, in addition to MALDI-TOF confirmation (data not shown) as described previously (Kelley et al., 2018). Personal identifiable information was removed before isolates were shipped to researchers at the University of Tennessee in accordance with the IRB protocol: UTK IRB-17-03683-XP. Only city and gender were collected as metadata. Once received, isolates were passaged on *Campylobacter-*selective media and grown for 48 h under microaerobic conditions at 37°C. This growth was harvested and used for genomic DNA extraction (below) and stocked in MH broth with 20% glycerol at -80C.

### Isolation of *Campylobacter* From Regional Samples

After incubation on selective media under the conditions described above, plates from each sample type were enumerated and 2-5 individual colonies were passaged onto the *Campylobacter-*selective media as described above and incubated for another 48 h under microaerobic conditions at 37°C. Resulting growth was harvested and used for both genomic DNA extraction (below) and stocked in MH broth with 20% glycerol at −80°C.

### Genomic DNA Preparation and PCR-Based Identification

Genomic DNA was obtained from growth of isolated colonies following the protocol described previously (Kelley et al., 2018). Briefly, growth was resuspended in sterile genomic lysis buffer (50 mM Tris Base - pH7.5, 50 mM EDTA, 1% SDS, 10 mM NaCl) before adding protein precipitation solution (Promega – A795A). Following DNA precipitation, the pellet was dried before resuspension in 100 μl ultra-pure water. Genomic DNA was stored at −20°C. Designation of samples as either *C. jejuni* or *C. coli* was conducted via PCR with primers that specifically amplify either *mapA* (Forward-TCAATGCAGTTCTTGTGAAA; Reverse-TTCAGAGATTAAACTAGCTGC) or *ceuE* (Forward–ATGAAAAAATATTTAGTTTTTGCA; Reverse-ATTTTATTATTTGTAGCAGCG), respectively under the following conditions: 95°C-5min; 50°C - 30 sec, 45°C - 30 sec, 72°C - 1min for 30 cycles; 72°C - 7 min (Gonzalez et al., 1997; Dekker, 2016). Resulting amplicons were imaged using a 1.0% agarose gel stained with ethidium bromide to check for a band of the corresponding sizes: *mapA*-550 bp or *ceuE*-893 bp.

### Preparation for Whole-Genome Sequencing

Isolates confirmed as *C. jejuni* were utilized for WGS. Each sample was RNase-treated by incubating 44 μl genomic DNA with 5 μl buffer and 1 μl RNase (Invitrogen – AM2294) for 1 h at 37°C before heat inactivating at 70°C for 20 min. The resulting RNase-treated DNA was cleaned using a Zymo Genomic DNA Clean and Concentrate Kit (D4011) following the manufacturer’s instructions. Genomic DNA sample concentrations were quantified on a NanoDrop 2000 spectrophotometer and visualized on a 1.0% agarose gel to confirm the presence of intact genomic DNA. Samples were aliquoted in nuclease-free 96-well plates and shipped to the Center for Genomics and Bioinformatics at Indiana University for WGS^1^.

### Whole-Genome Sequencing

Library preparation, multiplexing, and barcoding was conducted utilizing NEXTflex kits (PerkinElmer) following the manufacturer’s protocol (Perkinelmer-Appliedgenomics.com, 2020). DNA concentrations were obtained on a Qubit3 fluorometer before running on a 2200 TapeStation bioanalyzer. Sequencing was performed utilizing the Illumina NextSeq 500 platform with 150 × 150 paired end reads. The resulting paired-end reads were demultiplexed using bcl2fastq software (Support.Illumina.com, 2020). Resulting reads were accessed by researchers at Oak Ridge National Laboratory for genome assembly, annotation, and further bioinformatic analyses.

### Obtaining Reads From GenomeTrakr Database

After identifying the sampling period and region of interest, raw reads for 87 isolates deposited in the GenomeTrakr database were accessed and downloaded to incorporate into the network analysis. The accession numbers for reads used in the study can be found in Table 2.

### Bioinformatic Analysis of *C. jejuni* Genomes

The initial analysis of the resulting sequence reads and reads obtained from the GenomeTrakr database was performed as previously described (Kelley et al., 2018). Briefly, read quality for each sample was analyzed and adapter sequences were trimmed using Atropos (Didion et al., 2017). Genomes were *de novo* assembled from the remaining paired-end sequences using the up-to-date version (3.12.0) of SPAdes (Bankevich et al., 2012). For all genomes, quality was assessed using CheckM and low quality genome assemblies were removed (Parks et al., 2015). The Prokka genome annotation software package was utilized to predict protein-coding genes (Prodigal) and non-coding RNA genes (RNAmmer, tRNAscan-SE) (Lowe and Eddy, 1997; Lagesen et al., 2007; Hyatt et al., 2010; Seemann, 2014). Predicted proteins were annotated using Prokka and hmmscan (Finn et al., 2011) and were clustered into orthologous groups and designated as either belonging to the pan or core genomes using PIRATE (Bayliss et al., 2019). For each genome, a quantitative matrix was constructed and imported into Cytoscape for network analysis with a threshold of 0.93 (Shannon et al., 2003). Community clustering was then performed at 0.93 threshold to visualize distinct community groups (Newman, 2004; Su et al., 2010).

To compare the network-based analysis to previously established pipelines, we performed SNP calling using a CFSAN-based workflow with a minimal alternative allele frequency threshold of 90%, (Peerj.com, 2020). The following parameters were applied to assembled genomes – COV: 10, Rel. COV: 10%, SNP quality 30, MQ: 25, *Z*-score: 1.96, and distance between SNPs: 10. The *C. jejuni* 81-176_G1_B7 genome was used as a reference for the SNP analysis and tree assembly. The SNP tree was then visualized in iTOL (Letunic and Bork, 2016). In addition, assembled genomes were also assigned to ST clonal complexes for comparison. This was done by querying each assembled genome against the *Campylobacter* PubMLST database (Pubmlst.org, 2020).

### Antibiotic Susceptibility Testing of *C. jejuni* Isolates

Isolates used for susceptibility testing were cultured on MH plates containing trimethoprim (TMP) at 10 μg/ml and incubated under microaerobic conditions for 48 h at 37°C. Growth was harvested into 500 μl MH broth and a sterile cotton swab was used to spread each suspension on a large, 14 cm MH agar plate. Using the standard Kirby-Bauer method, Oxoid brand antibiotic disks of the following antibiotics and concentrations: Amoxycillin/Clavulanic Acid (30 μg), Ampicillin (10 μg), Azithromycin (15 μg), Ceftriaxone (30 μg), Cephazolin (30 μg), Ciprofloxacin (5 μg), Doxycycline (30 μg), Erythromycin (15 μg), Gentamicin (10 μg), Levofloxacin (5 μg), Meropenem (10 μg), and Tetracycline (30 μg), were dispensed onto each plate and incubated for 48–72 h under microaerobic conditions before zones of inhibition were measured. Measurements for individual antibiotics across all isolates were averaged and measurements for each isolate were subtracted from the average to determine strains that were more sensitive and more resistant in relation to the calculated average for each antibiotic.

## Results

### Sample Collection and *Campylobacter* Isolation

**FIGURE 1:**
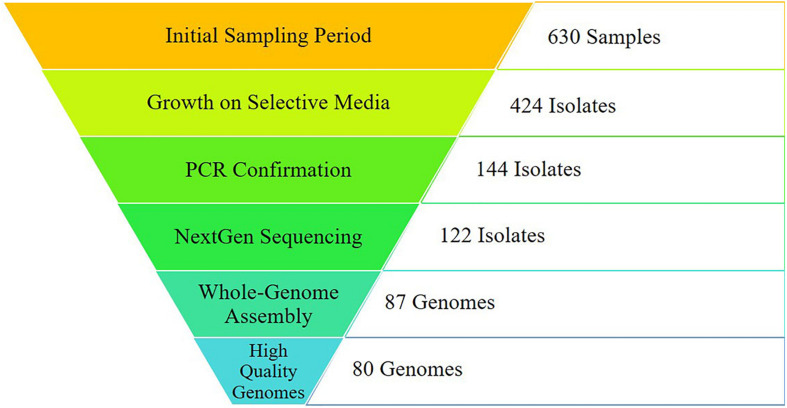
Sampling results from initial sampling to final analyses. Initial sampling was conducted across the region of East Tennessee from October 2016- October 2018, including environmental and human samples. *Campylobacter*-specific (CS) media was used for selective plating. Isolates were confirmed as either *C. jejuni* or *C. coli* utilizing primers specific for each. Quality control was conducted on raw sequencing reads before *de novo* assembly. Genomes assembling with 95% completeness or higher were utilized for downstream analyses. A total of 167 genomes (80 from this study and 87 from GenomeTrakr) were used for analysis.

Through the combined efforts of our group, veterinarians at the University of Tennessee College of Veterinary Medicine, and the Tennessee Department of Health, a total of 630 samples were collected (Figure 1). Of these, 293 fecal samples were collected from various animal sources, including alpaca, camel, cat, chicken, cow, dog, falcon, goat, goose, horse, pig, raccoon, sheep, snake, and zebra throughout the sampling period (Table 1). During the same period, 65 food samples were collected from local farmer’s markets and grocery stores, including vegetables (unwashed potatoes, kale leaves, and carrots), fruits (unwashed berries, citrus fruit, apples, bananas, peaches, and plums), and raw meats (chicken, pork, and beef). Sampling of local commercial meat processing plants resulted in fecal samples from cattle and pigs, all collected post-slaughter, included in the total listed above. Local surface water was also collected throughout the sampling period, with 165 samples collected from the banks of local running rivers and small streams, including the Tennessee River, the French Broad River, the Holston River, and lower tributaries.

**TABLE 1:** Breakdown of sampling numbers by source type, percent of total for each source type, PCR-confirmed *C. jejuni* samples, isolates submitted for sequencing, and total sequenced.

Source	# of samples (% of total)	Confirmed *C. jejuni* via PCR (% of total samples)	Submitted for sequencing	Sequenced
Alpaca	9 (1.43)	0/9 (0)	0	0
Camel	2 (0.32)	0/2 (0)	0	0
Cat	9 (1.43)	0/9 (0)	0	0
Chicken	53 (8.41)	15/53 (28.3)	11	8
Cow	79 (12.54)	16/79 (20.2)	16	14
Dog	36 (5.71)	1/36 (2.7)	1	1
Falcon	1 (0.16)	1/1 (100)	1	1
Food (non-meat)	44 (6.98)	3/44 (6.8)	3	3
Food (meat)	21 (3.33)	0/21 (0)	0	0
Goat	7 (1.11)	0/7 (0)	0	0
Goose	5 (0.79)	1/5 (20)	1	1
Horse	52 (8.25)	7/52 (13.4)	3	3
Human	76 (12.06)	71/76 (93.4)	70	70
Pig	36 (5.87)	7/36 (19.4)	7	7
Raccoon	1 (0.16)	0/1 (0)	0	0
Sheep	30 (4.76)	6/30 (20)	5	5
Snake	2 (0.32)	0/2 (0)	0	0
Water	165 (26.19)	16/165 (9.6)	9	9
Zebra	1 (0.16)	0/1 (0)	0	0
Totals:	630	144/630 (22.8)	127	122

Successful isolation and PCR-confirmation of *C. jejuni* varied greatly by source. Samples yielded PCR-positive isolates as follows: chickens-15, cattle-16, sheep-6, horse-7, pig-7, dog-1, falcon-1, goose-1, non-meat food-3, and water-16. We did not obtain PCR-confirmed *C. jejuni* isolates from alpaca, camel, cat, goat, raccoon, snake, and zebra samples. Human isolates were collected by the Tennessee Department of Health during the previously described sampling period. Of the 76 human isolates received by our group, 71 were *C. jejuni*, 4 were *C. coli*, and a single isolate was *C. hyointestinalis*. Overall, a single PCR-confirmed *C. jejuni* isolate from each sample was prepared and submitted for whole-genome sequencing and downstream analyses. Of the 127 genomes submitted for sequencing, 122 samples (Chicken-8, Cow-14, Dog-1, Falcon-1, Non-meat food-3, Goose-1, Horse-3, Human-70, Pig-7, Sheep-5, Water-9) passed quality control standards employed by the sequencing facility (DNA quality/quantity) and resulted in high quality reads. At least 50 million reads were generated for each isolate, representing 100x coverage of each *C. jejuni* genome (∼1.7 Mb).

### Whole-Genome Assembly and Quality Filtering

**FIGURE 2:**
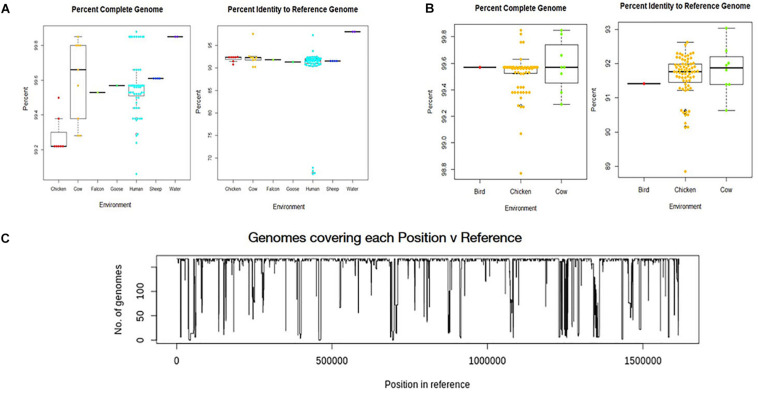
Analysis of genome assemblies for completeness, identity to reference, and coverage. **(A)** Box and whisker plot of genome completeness and percent identity to the reference were compiled for each source type for samples collected in this study. **(B)** Box and whisker plots were also compiled for GenomeTrakr genomes. Completeness across all assembled genomes ranged from 98.0% to above 99.8% and assembled genomes had an average alignment of 92% to the reference genome (*C. jejuni* 81-176_G1_B7). used for this study. **(C)** Coverage of each position in the reference genome by the genomes analyzed.

All environmental genomes utilized for the whole genome analyses are between 98.0% and 99.9% complete as determined by analysis with CheckM (Figures 2A,B) (Parks et al., 2015). The range of completeness for the environmental genomes varied between isolate source, with human isolates demonstrating a range in completeness of 99.0% - > 99.8%. The range of completeness for chicken isolates was 98.0% – 99.8% across all genomes analyzed, while cattle isolates presented a range in completeness (99.3% – 99.875%) similar to that of humans. Only genomes mapping to a reference genome (*C. jejuni* 81-176_G1_B7) with 90% identity or above to ensure species identity were utilized for the study. Overall, the genomes from various sources mapped to the reference genome at an average of 92% identity, with the exception of the water samples. The genomes from water isolates that clustered with an identity close to 98% of the reference genome. Interestingly, there was some variability in percent identity of human isolates to the reference, with several close to 70% identity to the reference genome (Figure 2A) and therefore excluded from further analyses. The number of genomes covering each position in the reference sequence was also relatively high, although some positions in the reference were covered by only a few genomes suggesting more novel regions in the reference genome (Figure 2C).

### Core and Pan Genome Assembly and Analysis

**FIGURE 3:**
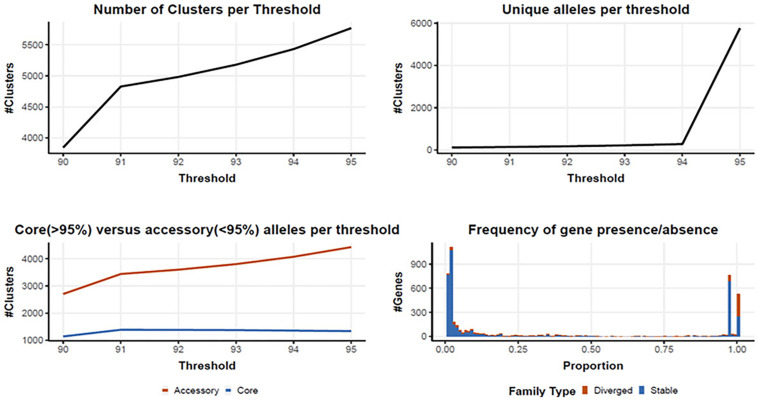
Core and pan genome assembly and gene family distribution and identification of new genes. One hundred and sixty seven genomes were used to identify a core and pan genome. There were 4710 gene families identified in the pangenome, with 225 gene families containing greater than one allele at the 90–95% threshold. The core genome is comprised of 1,384 gene families, with 3326 accessory gene families.

Following quality filtering and genome assembly, 167 high quality genomes were used to construct an analytical pipeline that was used to define both the core and pan genome, which were based on the presence of predicted gene families (Figure 3). In all, there were 4710 gene families identified in the 167 *C. jejuni* genomes analyzed by PIRATE with 225 gene families containing greater than one allele at the 90–95% threshold. The pangenome of *C. jejuni* comprised 4710 gene families of which 1384 were classified as core (defined as genes found in >95% of the genomes) and 3326 accessory genes. The large number of accessory genes identified in the pan genome analysis indicates *Campylobacter* isolates have remarkable genetic variability and likely contribute to their broad host distribution

### SNP and Network Analysis for Comparison of Assembled Genomes

**FIGURE 4:**
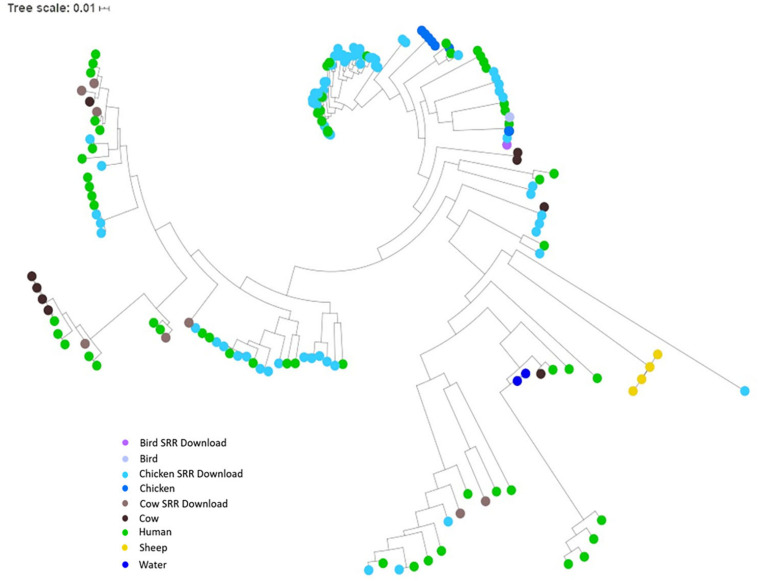
SNP tree of environmental isolates demonstrating relatedness. A tree of relatedness was created using 167 assembled genomes, organized by source, utilizing *C. jejuni* 81-176_G1_B7 as a reference. Colors denote sample source, with GenomeTrakr genomes identified as SRR Download.

**FIGURE 5:**
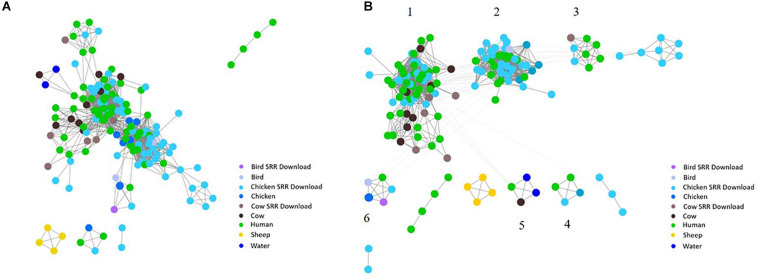
Network analysis with assembled genomes. A network analysis of 167 genomes and the coding regions across all genomes. Distance between nodes is arbitrary, but edges connecting individual nodes indicate these meet or surpass a threshold of 0.93 similarity across the entire coding portion of the two genomes. **(A)** The initial unclustered genomes falling above the threshold of 0.93 similarity. **(B)** The same network with community clustering applied. GenomeTrakr downloads are labeled SRR Download.

To investigate the potential of whole genome comparison as a means for *Campylobacter* source-tracking, genomes underwent a SNP analysis to determine the potential relatedness of various isolates. A tree of relatedness was produced of all isolates using *C. jejuni* 81-176_G1_B7 as a reference genome (Figure 4). Isolate source is denoted by color. We also employed a non-reference-based approach for whole genome comparisons using network analysis. The genomes from this study and GenomeTrakr were analyzed by comparing coding regions to produce a network for visualization of relatedness (Figure 5). While the distance between nodes is arbitrary, lines connecting individual nodes indicate they meet or exceed a threshold of 0.93 across the entire coding region of the two genomes. The network was further characterized by clustering genomes with community clustering (Figure 5B). Community clustered genomes reveals human isolates cluster with other human isolates, but also cluster with those from other sources including cows/chickens (Cluster 1 and 3), cows/water (Cluster 5), and chickens/other birds (Clusters 2, 4, and 6).

### ST Assignments of Isolates Based on Whole-Genomes

The whole genome of each isolate identified within clusters of the network analysis were queried against the PubMLST online database of *Campylobacter* strains. Isolates Human-40, Cow-2, Chicken(meat)-6, Chicken(live)-7, Chicken (meat)-12, Chicken(meat)-14, Chicken(meat)-38, Cow-16, Cow-18, Chicken(meat)-58, Chicken(meat)-59, Chicken(meat)-61, and Cow-22 were not assigned to a clonal complex, but were assigned to ST groups, as shown in Table 2. Additionally, five genomes did not match to any previously identified ST group or clonal complex. ST-353 was the most common assignment across all the genomes, especially the GenomeTrakr isolates (Table 2).

**TABLE 2:** ST assignments for each isolate identified in the cluster analysis.

Isolate source	Sample name	NCBI accession #	ST-assignment	Clonal complex	Sample source
This study	Human-1	SRR12633998	831	ST-828	Human feces
This study	Human-2	SRR12633999	61	ST-61	Human feces
This study	Human-3	SRR12634000	122	ST-206	Human feces
This study	Human-4	SRR12634001	2132	ST-353	Human feces
This study	Human-5	SRR12634003	50	ST-21	Human feces
This study	Cow-6	SRR12625354	61	ST-61	Cattle feces
This study	Human-6	SRR12634004	767	ST-45	Human feces
This study	Human-7	SRR12634005	48	ST-48	Human feces
This study	Human-8	SRR12634006	21	ST-21	Human feces
This study	Human-9	SRR12634007	61	ST-61	Human feces
This study	Human-10	SRR12634008	902	ST-828	Human feces
This study	Human-11	SRR12634009	508	ST-508	Human feces
This study	Human-12	SRR12634010	583	ST-45	Human feces
This study	Human-13	SRR12634011	21	ST-21	Human feces
This study	Human-14	SRR12634012	353	ST-353	Human feces
This study	Human-15	SRR12634014	10068	ST-48	Human feces
This study	Human-16	SRR12634015	1212	ST-607	Human feces
This study	Human-17	SRR12634016	48	ST-48	Human feces
This study	Human-18	SRR12634017	52	ST-52	Human feces
This study	Cow-7	SRR12633996	61	ST-61	Cattle feces
This study	Human-19	SRR12634018	52	ST-52	Human feces
This study	Human-20	SRR12634019	267	ST-283	Human feces
This study	Human-21	SRR12634020	607	ST-607	Human feces
This study	Human-22	SRR12634021	353	ST-353	Human feces
This study	Human-23	SRR12634022	354	ST-354	Human feces
This study	Human-24	SRR12634023	6645	ST-49	Human feces
This study	Human-25	SRR12634025	50	ST-21	Human feces
This study	Human-26	SRR12634026	4559	ST-42	Human feces
This study	Human-27	SRR12634027			Human feces
This study	Human-28	SRR12634028	353	ST-353	Human feces
This study	Human-29	SRR12634029	1244	ST-61	Human feces
This study	Human-30	SRR12634030	353	ST-353	Human feces
This study	Cow-8	SRR12633997	61	ST-61	Cattle feces
This study	Human-31	SRR12634031	48	ST-48	Human feces
This study	Human-32	SRR12634032	222	ST-206	Human feces
This study	Human-33	SRR12634033	353	ST-353	Human feces
This study	Human-34	SRR12634034	21	ST-21	Human feces
This study	Human-35	SRR12634036	354	ST-354	Human feces
This study	Human-36	SRR12634037	353	ST-353	Human feces
This study	Human-37	SRR12634038	52	ST-52	Human feces
This study	Human-38	SRR12634039			Human feces
This study	Human-39	SRR12634040	829	ST-828	Human feces
This study	Human-40	SRR12634041	6091		Human feces
This study	Human-41	SRR12634042	137	ST-45	Human feces
This study	Human-42	SRR12634043	353	ST-353	Human feces
This study	Human-43	SRR12634044	52	ST-52	Human feces
This study	Human-44	SRR12634045	50	ST-21	Human feces
This study	Human-45	SRR12634047	353	ST-353	Human feces
This study	Human-46	SRR12634048	10533	ST-49	Human feces
This study	Human-47	SRR12634049	22	ST-22	Human feces
This study	Human-48	SRR12634050	1244	ST-61	Human feces
This study	Human-49	SRR12634051	353	ST-353	Human feces
This study	Human-50	SRR12634052	50	ST-21	Human feces
This study	Human-51	SRR12634053			Human feces
This study	Human-52	SRR12634054	2862	ST-21	Human feces
This study	Human-53	SRR12634055	5453	ST-179	Human feces
This study	Human-54	SRR12634056	2862	ST-21	Human feces
This study	Human-55	SRR12634058	5862	ST-21	Human feces
This study	Chicken (live)-2	SRR12634059	4489	ST-353	Chicken feces
This study	Chicken (live)-3	SRR12634060	4489	ST-353	Chicken feces
This study	Chicken (live)-6	SRR12634061	4489	ST-353	Chicken feces
This study	Non-chicken Bird-1	SRR12634062			Goose feces
This study	Non-chicken Bird-2	SRR12634063	353	ST-353	Falcon feces
This study	Sheep-1	SRR12634064	265		Sheep feces
This study	Sheep-2	SRR12634065	265		Sheep feces
This study	Sheep-3	SRR12634066	265		Sheep feces
This study	Sheep-4	SRR12634067	265		Sheep feces
This study	Water-1	SRR12634069	604	ST-42	Holston River
This study	Human-56	SRR12634070	1068	ST-828	Human feces
This study	Human-57	SRR12634071	8	ST-21	Human feces
This study	Chicken (live)-8	SRR12634072	460	ST-460	Chicken feces
This study	Cow-9	SRR12634073	459	ST-42	Cattle feces
This study	Human-58	SRR12634074	460	ST-460	Human feces
This study	Human-59	SRR12634075	61	ST-61	Human feces
This study	Human-60	SRR12634076	3510	ST-353	Human feces
This study	Human-61	SRR12634077	45	ST-45	Human feces
This study	Human-62	SRR12634078	806	ST-21	Human feces
This study	Human-63	SRR12633995	806	ST-21	Human feces
This study	Cow-1	SRR12634002	982	ST-21	Cattle feces
This study	Cow-2	SRR12634013	922		Cattle feces
This study	Cow-3	SRR12634024	61	ST-61	Cattle feces
This study	Cow-4	SRR12634035	929	ST-257	Cattle feces
This study	Cow-5	SRR12634046	929	ST-257	Cattle feces
This study	Chicken (live)-1	SRR12634057	4489	ST-353	Chicken feces
This study	Chicken (live)-4	SRR12634068	1212	ST-607	Chicken feces
This study	Chicken (live)-5	SRR12634079	4489	ST-353	Chicken feces
This study	Water-2	SRR12634080	604	ST-42	Tennessee River
Genome Trakr	Chicken (meat)-1	SRR7697949	8065	ST-353	Chicken meat
Genome Trakr	Chicken (meat)-2	SRR7697950	3510	ST-353	Chicken meat
Genome Trakr	Chicken (meat)-3	SRR7698002	10738	ST-353	Chicken meat
Genome Trakr	Chicken (meat)-4	SRR7698003	50	ST-21	Chicken meat
Genome Trakr	Chicken (meat)-5	SRR7698092	48	ST-48	Chicken meat
Genome Trakr	Chicken (meat)-6	SRR7795493	56		Chicken meat
Genome Trakr	Cow-10	SRR5858786	38	ST-48	Cattle feces
Genome Trakr	Chicken (live)-7	SRR7820308	56		Chicken feces
Genome Trakr	Chicken (meat)-7	SRR6224682	267	ST-283	Chicken meat
Genome Trakr	Cow-11	SRR5271464	10578	ST-353	Cattle feces
Genome Trakr	Cow-12	SRR4453678	806	ST-21	Cattle feces
Genome Trakr	Chicken (meat)-8	SRR6108167	9451	ST-353	Chicken meat
Genome Trakr	Chicken (meat)-9	SRR6108171	3736	ST-353	Chicken meat
Genome Trakr	Chicken (meat)-10	SRR6108172	3736	ST-353	Chicken meat
Genome Trakr	Chicken (meat)-11	SRR6108173	353	ST-353	Chicken meat
Genome Trakr	Chicken (meat)-12	SRR6108174	56		Chicken meat
Genome Trakr	Chicken (meat)-13	SRR6108175	939	ST-353	Chicken meat
Genome Trakr	Chicken (meat)-14	SRR6108176	940		Chicken meat
Genome Trakr	Chicken (meat)-15	SRR6108233	21	ST-21	Chicken meat
Genome Trakr	Chicken (meat)-16	SRR6108235	429	ST-48	Chicken meat
Genome Trakr	Chicken (meat)-17	SRR6108238	353	ST-353	Chicken meat
Genome Trakr	Chicken (meat)-18	SRR6108240	48	ST-48	Chicken meat
Genome Trakr	Chicken (meat)-19	SRR6108241	48	ST-48	Chicken meat
Genome Trakr	Chicken (meat)-20	SRR6108242	353	ST-353	Chicken meat
Genome Trakr	Chicken (meat)-21	SRR6108318	353	ST-353	Chicken meat
Genome Trakr	Chicken (meat)-22	SRR6108321	3510	ST-353	Chicken meat
Genome Trakr	Chicken (meat)-23	SRR6108322	3510	ST-353	Chicken meat
Genome Trakr	Chicken (meat)-24	SRR6108388	3510	ST-353	Chicken meat
Genome Trakr	Chicken (meat)-25	SRR6108391	3515	ST-353	Chicken meat
Genome Trakr	Chicken (meat)-26	SRR6108394	45	ST-45	Chicken meat
Genome Trakr	Chicken (meat)-27	SRR6108425	460	ST-460	Chicken meat
Genome Trakr	Chicken (meat)-28	SRR6108427	9062	ST-353	Chicken meat
Genome Trakr	Non-chicken bird (meat)-1	SRR6108429	460	ST-460	Turkey meat
Genome Trakr	Chicken (meat)-29	SRR6108542	939	ST-353	Chicken meat
Genome Trakr	Chicken (meat)-30	SRR6108543	11348	ST-353	Chicken meat
Genome Trakr	Chicken (meat)-31	SRR6108546	4370	ST-353	Chicken meat
Genome Trakr	Chicken (meat)-32	SRR6108547	3735	ST-353	Chicken meat
Genome Trakr	Chicken (meat)-33	SRR6108549	4370	ST-353	Chicken meat
Genome Trakr	Cow-13	SRR5925264	982	ST-21	Cattle feces
Genome Trakr	Chicken (meat)-34	SRR6345309	51	ST-443	Chicken meat
Genome Trakr	Chicken (meat)-35	SRR5750559	137	ST-45	Chicken meat
Genome Trakr	Chicken (meat)-36	SRR6158111	50	ST-21	Chicken meat
Genome Trakr	Chicken (meat)-37	SRR7888728	48	ST-48	Chicken meat
Genome Trakr	Chicken (meat)-38	SRR7888755	10412		Chicken meat
Genome Trakr	Chicken (meat)-39	SRR5982360	3510	ST-353	Chicken meat
Genome Trakr	Chicken (meat)-40	SRR5217510	3595	ST-48	Chicken meat
Genome Trakr	Chicken (meat)-41	SRR5217516	3510	ST-353	Chicken meat
Genome Trakr	Chicken (meat)-42	SRR5217524	50	ST-21	Chicken meat
Genome Trakr	Chicken (meat)-43	SRR5217529	3595	ST-48	Chicken meat
Genome Trakr	Cow-14	SRR5821345	982	ST-21	Cattle feces
Genome Trakr	Cow-15	SRR5683959	48	ST-48	Cattle feces
Genome Trakr	Chicken (meat)-44	SRR6504977	464	ST-464	Chicken meat
Genome Trakr	Chicken (meat)-45	SRR6498666	51	ST-443	Chicken meat
Genome Trakr	Chicken (meat)-46	SRR5414469	452	ST-353	Chicken meat
Genome Trakr	Chicken (meat)-47	SRR5414473	48	ST-48	Chicken meat
Genome Trakr	Chicken (meat)-48	SRR5414474	475	ST-48	Chicken meat
Genome Trakr	Chicken (meat)-49	SRR5414637	51	ST-443	Chicken meat
Genome Trakr	Chicken (meat)-50	SRR5414638	353	ST-353	Chicken meat
Genome Trakr	Chicken (meat)-51	SRR5414815	1287	ST-1287	Chicken meat
Genome Trakr	Chicken (meat)-52	SRR5414818	353	ST-353	Chicken meat
Genome Trakr	Chicken (meat)-53	SRR5414820	353	ST-353	Chicken meat
Genome Trakr	Chicken (meat)-54	SRR5414822	353	ST-353	Chicken meat
Genome Trakr	Chicken (meat)-55	SRR5414913	597	ST-21	Chicken meat
Genome Trakr	Chicken (meat)-56	SRR5414391	3510	ST-353	Chicken meat
Genome Trakr	Chicken (meat)-57	SRR5414394	3515	ST-353	Chicken meat
Genome Trakr	Cow-16	SRR5448586			Cattle feces
Genome Trakr	Cow-17	SRR5423637	21	ST-21	Cattle feces
Genome Trakr	Cow-18	SRR5164546	922		Cattle feces
Genome Trakr	Cow-19	SRR5164549	61	ST-61	Cattle feces
Genome Trakr	Cow-20	SRR5164557	45	ST-45	Cattle feces
Genome Trakr	Chicken (meat)-58	SRR7503412	922		Chicken meat
Genome Trakr	Chicken (meat)-59	SRR7503417	922		Chicken meat
Genome Trakr	Chicken (meat)-60	SRR7503418	8768	ST-353	Chicken meat
Genome Trakr	Chicken (meat)-61	SRR7503419	922		Chicken meat
Genome Trakr	Chicken (meat)-62	SRR7503542	353	ST-353	Chicken meat
Genome Trakr	Chicken (meat)-63	SRR7503553	354	ST-354	Chicken meat
Genome Trakr	Chicken (meat)-64	SRR7465553	939	ST-353	Chicken meat
Genome Trakr	Chicken (meat)-65	SRR7406245	607	ST-607	Chicken meat
Genome Trakr	Chicken (meat)-66	SRR7903297	464	ST-464	Chicken meat
Genome Trakr	Chicken (meat)-67	SRR7903352	353	ST-353	Chicken meat
Genome Trakr	Cow-21	SRR7521557	9111	ST-21	Cattle feces
Genome Trakr	Chicken (meat)-68	SRR7525351	353	ST-353	Chicken meat
Genome Trakr	Chicken (meat)-69	SRR7503644	452	ST-353	Chicken meat
Genome Trakr	Chicken (meat)-70	SRR7503647	452	ST-353	Chicken meat
Genome Trakr	Chicken (meat)-71	SRR7503761	658	ST-658	Chicken meat
Genome Trakr	Chicken (meat)-72	SRR6888982	51	ST-443	Chicken meat
Genome Trakr	Cow-22	SRR5043248	10501		Cattle feces
Genome Trakr	Chicken (meat)-73	SRR5043956	3510	ST-353	Chicken meat

*Whole genomes were queried against the online Campylobacter database to identify the ST and clonal complex assignment for each isolate.*

### Antibiotic Susceptibility Testing

**FIGURE 6:**
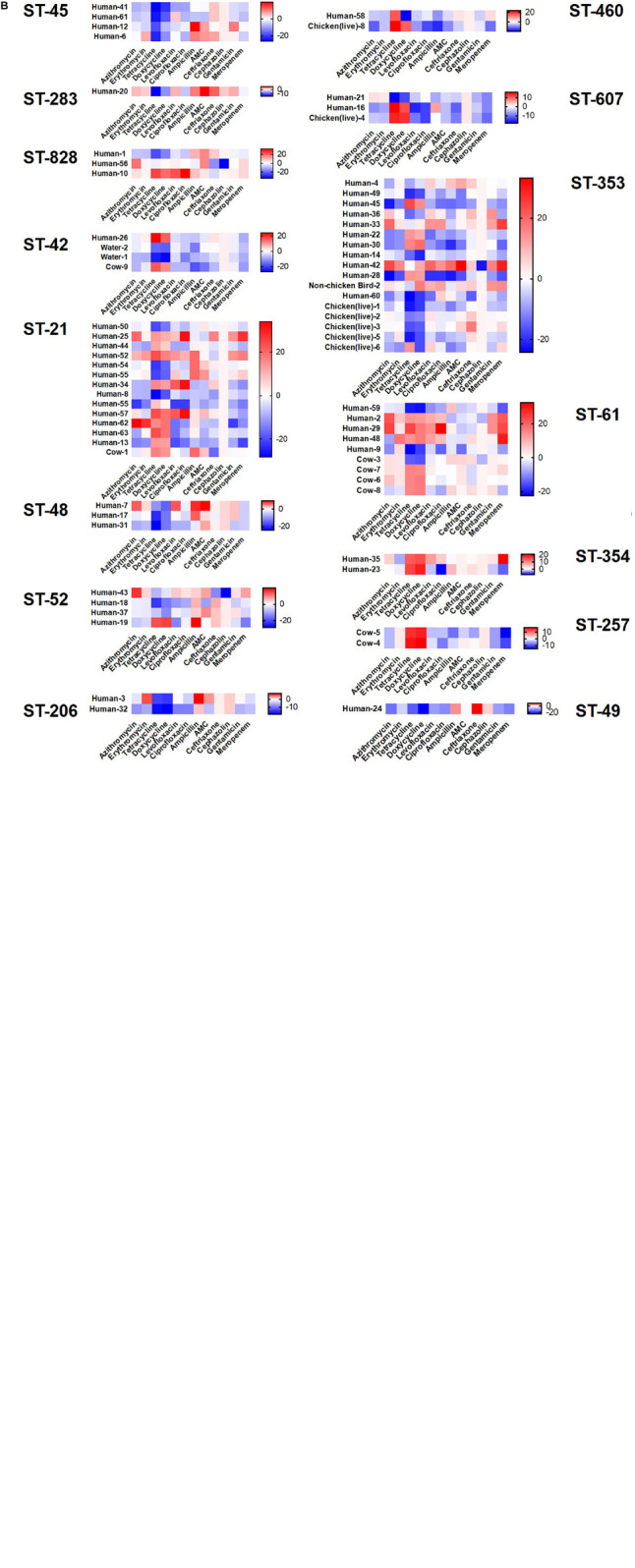
Antibiotic susceptibility testing results according to cluster. Antibiotic susceptibility to several classes of antibiotics were compared across source type **(A)** and ST grouping **(B)** by subtracting measurements for each isolate from the overall average for each antibiotic. Positive numbers (red) indicate isolates less susceptible to the corresponding antibiotic, while negative numbers (blue) indicate isolates more susceptible. No obvious patterns were distinguishable across the groupings despite extensive heterogeneity.

We assayed isolates identified in this study against two antibiotics each from six different antibiotic classes to investigate phenotypic differences (Figure 6). The greatest amount of variability for a single antibiotic class appears for tetracycline and doxycycline which display a broader spectrum of sensitivity across all the isolates, regardless of cluster, when compared to results for the other antibiotics. Human isolates displayed the greatest variability in antibiotic sensitivity across the antibiotics assayed. With a few exceptions, cattle isolates demonstrated a pattern of lower overall susceptibility across the spectrum of antibiotics assayed when compared to other isolates, while isolates from chickens displayed increased susceptibility to the majority of antibiotics when compared to other isolates (Figure 6A). When separated based on ST assignment, the overall broader sensitivity to tetracycline and doxycycline can be observed as well (Figure 6B).

## Discussion

To investigate whether a whole genome-based approach is a viable method for source-tracking human *Campylobacter* infections in a geographically and temporally restricted manner, we conducted active surveillance of agricultural and environmental sources in eastern Tennessee between October 2016 and October 2018. Confirmed *C. jejuni* isolates obtained during the sampling period were subjected to whole-genome sequencing and bioinformatically compared to clinical isolates deposited with the Tennessee Department of Health and GenomeTrakr sequences from the same geographical area during the indicated sampling period. Historically, East Tennessee has a higher incidence of *C. jejuni* infections (8–11 per 100,000 persons) when compared to the rest of the state (5–7 per 100,000 persons) (Weisent et al., 2011; Tn.gov, 2020). Despite this incidence, East Tennessee is home to relatively few poultry farms and other agricultural operations, although there is some variability between counties in the region (Nass.Usda.Gov,, 2020). This observation is particularly pronounced in Knox County, where numerous human isolates were collected, but where agricultural production is limited. In addition to agricultural operations, the region also contains numerous rivers, streams, and tributaries that experience flooding throughout the year, and may lead to contamination of groundwater or recreational waters, increasing the risk of human exposure. During the study period, East Tennessee was also impacted by a *C. jejuni* outbreak that was attributed to puppies sold by a large pet store chain (Montgomery et al., 2018). Taken together, these factors suggested that *C. jejuni* infections in the region may not be due solely to the consumption of undercooked or contaminated poultry meat, but may also be the result of interactions with other infected animals or contaminated water sources.

Our study is unique in that the analyzed strains were isolated from the same region during the same period, which provided a “real world” scenario of sampling for source-tracking using whole-genome sequencing in a region that had not been previously investigated. Of over 600 samples collected, we were able to generate 80 complete, high quality genomes for our analyses. With less than one quarter (∼12.7%) of the collected samples resulting in a high quality genome, successful surveillance of a region like the one described in this study would likely be time consuming and costly. This was a considerable obstacle in creating an adequate set of reference isolates within our geographical area, but was addressed by supplementing with genomes submitted to an online database from the same region and time period, which underscores the importance of efforts like the GenomeTrakr program. In addition to the relatively low rate of high quality genome generation, several samples yielded growth on *Campylobacter*-specific media, but could not be identified as either *C. jejuni* or *C. coli* by PCR. Such a result indicates that other *Campylobacter* species may be present in the environmental sources, which may be worth further investigation in the future.

Assembly of the *C. jejuni* genomes from raw sequence reads was successful and utilized a high threshold for completeness, ensuring that only the most informative genomes were used. It is possible to lower the threshold of completeness in order to include more genomes that are less complete, but doing so may negatively affect the results and skew downstream analyses. Although we were able to identify a core and pan genome using the data provided from the combined 167 genomes, utilizing a larger set of genomes would ensure all genes in the core genome are identified, as well as all potential genes in the pan genome. In subsequent studies, the resulting pan genome could then be used to identify host-specific determinants, virulence factors that impact human campylobacteriosis severity, and bacterial factors that promote survival within different environments. Since *C. jejuni* isolates were the most commonly isolated species from human clinical samples, the *Campylobacter jejuni* 81-176_G1_B7 served as a reference strain to determine genome assembly quality. While the potential for identity bias should be kept in mind when selecting a reference genome for other types of analyses, the analyses described in this study avoid the introduction of these biases by only using the reference genome to ensure genomes are from *C. jejuni* before direct comparison of the genomes to each other.

The unique network analysis described in this study produced clusters of human and environmental isolates, suggesting whole genome comparisons may be a viable method for linking human infections to potential source types, although tracking to a specific site may not be possible. Interestingly, human isolates most frequently clustered with cattle and chicken isolates as indicated by the number of edges linking human isolates to those from cattle, chickens, or both. We believe the network analysis provides a visual representation of the similarities between isolates by denoting clusters that may not be indicated or obvious in the SNP dendrogram. Portraying the data in a way such as the network analysis also potentially increases the ability to identify clusters or isolates that should be examined more closely. This method could also prove useful since the threshold of similarity can be adjusted as necessary based on the organism of interest and the level of similarity desired for comparison.

The genetic variability between isolates from different sources led us to question whether phenotypic variability also exists between isolates and if patterns can be detected between sampling sources. This study utilized the Kirby-Bauer disk diffusion method to obtain preliminary phenotypic data by comparing the zones of inhibition for individual isolates to the overall average zone of inhibition for each antibiotic, allowing for intraspecies comparison across an array of antibiotics. Overall, a large amount of variability was easily detected between the isolates making the identification of phenotypic patterns challenging. Cattle isolates collected at random during routine checks of healthy animals on farms demonstrated lower overall susceptibility across the spectrum of antibiotics assayed. While no data was collected regarding antibiotic use on these farms, further epidemiological work may provide insights into potential links to antibiotic usage and susceptibility in the coordinating isolates. As a preliminary analysis focused on direct phenotypic comparisons, this analysis did not take into account minimum inhibitory concentration (MIC) data or clinical breakpoints for the antibiotics tested. However, the observed variability between isolates from the same source type indicate the potential for future work to investigate antibiotic susceptibility of environmental isolates in a more clinically relevant manner.

This work demonstrates the potential of whole-genome sequencing as a means of microbial source tracking for *C. jejuni*, but also identifies issues that must be addressed before this technique can be adequately utilized in an effective manner. WGS can provide a breadth of genomic information for comparison, but the sequencing quality and computing power necessary to conduct such in-depth analyses can be limiting factors. While the analyses described in this study would be useful for investigating the genomic relatedness of *Campylobacter* in the environment and the clinic, the ability to perform these in-depth analyses may not be realistic for groups without access to supercomputing facilities. Additionally, the level of surveillance necessary to maintain a current profile of *C. jejuni* genomic information from environmental sources in an accessible database would require continual sampling from numerous sources, which may also prove to be a limiting factor for state and federal health departments. Again, this need underscores the importance and continued support for resources like the FDA GenomeTrakr program.

As demonstrated by the network analyses, *Campylobacter* can be clustered by potential source, but the inherent genomic variability between individual isolates may make linking cases to a specific source challenging. Previous studies have demonstrated the benefits of whole-genome sequencing for comparing environmental and human isolates through the incorporation of sequences deposited in online databases (Wilson et al., 2008; Dearlove et al., 2016; Buchanan et al., 2017). By utilizing the GenomeTrakr database, we were able to increase the number of genomes used for our analyses and enhance the quality of the network. The resulting clusters support the conclusion that both chickens and cattle may serve as sources for human infections in East Tennessee, which has been observed in similar studies conducted in France and Germany (Rosner et al., 2017; Thépault et al., 2018a, b). Using this form of network analysis with a thorough examination of the corresponding epidemiological data could provide insight into risk factors leading to *C. jejuni* infections in East Tennessee.

Our study is the first to utilize WGS technology to preliminarily analyze isolates collected from a variety of sources in East Tennessee during a set sampling period and incorporate sequence information deposited in an online repository to compare to human isolates from the same region. Based on our results, we believe whole-genome sequencing is a beneficial technique that can provide an abundance of genomic data and source-tracking information. Additionally, the in-depth curation and analyses of epidemiological data by state and federal health agencies are necessary for source-tracking of human *Campylobacter* infections, along with concurrent surveillance of potential reservoirs throughout the region. While this may prove a major hurdle, we believe the implementation of WGS technology and our network analysis method can provide valuable information about the presence of environmental *C. jejuni* in regions like East Tennessee, and when compiled with epidemiological data, can aid in the identification of potential sources of human infection.

## Data Availability Statement

The datasets presented in this study can be found in GenBank under the BioProject number PRJNA644378 for samples collected in the study. NCBI accession numbers for all samples used in this study can be found in Table 2.

## Ethics Statement

The studies involving human participants were reviewed and approved by University of Tennessee Institutional Review Board. The ethics committee waived the requirement of written informed consent for participation. Ethical review and approval was not required for the animal study because the researchers were never in contact with animals from which samples were acquired. Samples from livestock were obtained from licensed DVMs during routine health exams. Written informed consent for participation was not obtained from the owners because Written informed consent was not required as these were indirect samples obtained during routine health exams. The researchers never had contact with the animals and no identifiable information was provided by the veterinarians.

## Author Contributions

BK, JE, DJ, and JJ conceived and designed the experiments. BK and JE performed the experiments and analyzed the data. AL aided in analysis of bioinformatic analyses. LS performed the epidemiological analysis. BK, JE, LS, and JJ wrote and revised the manuscript. All the authors have read and approved the final manuscript.

## Conflict of Interest

The authors declare that the research was conducted in the absence of any commercial or financial relationships that could be construed as a potential conflict of interest.

## Footnotes

1 https://cgb.indiana.edu/

## Supplementary Material

Supplementary Figure 1 Percentage identity of gene family at lowest threshold. Breakdown of percent identity of gene families at the lowest threshold. Identified gene families were classified as either fission/fusion, fission/fusion + multicopy, multicopy, or single copy.

Supplementary Table 1 Sampling location and date. Sampling source is listed for individual samples collected in this study. Sampling locations are listed by county in Tennessee, along with the collection date for each sample.
