# Supplementary figures and images for: Whole-Genome Sequencing and Bioinformatic Analysis of Environmental, Agricultural, and Human Campylobacter jejuni Isolates From East Tennessee
Source: Front Microbiol. 2020 Nov 5;11:571064. doi: 10.3389/fmicb.2020.571064 (PMC7674308; doi:10.3389/fmicb.2020.571064)

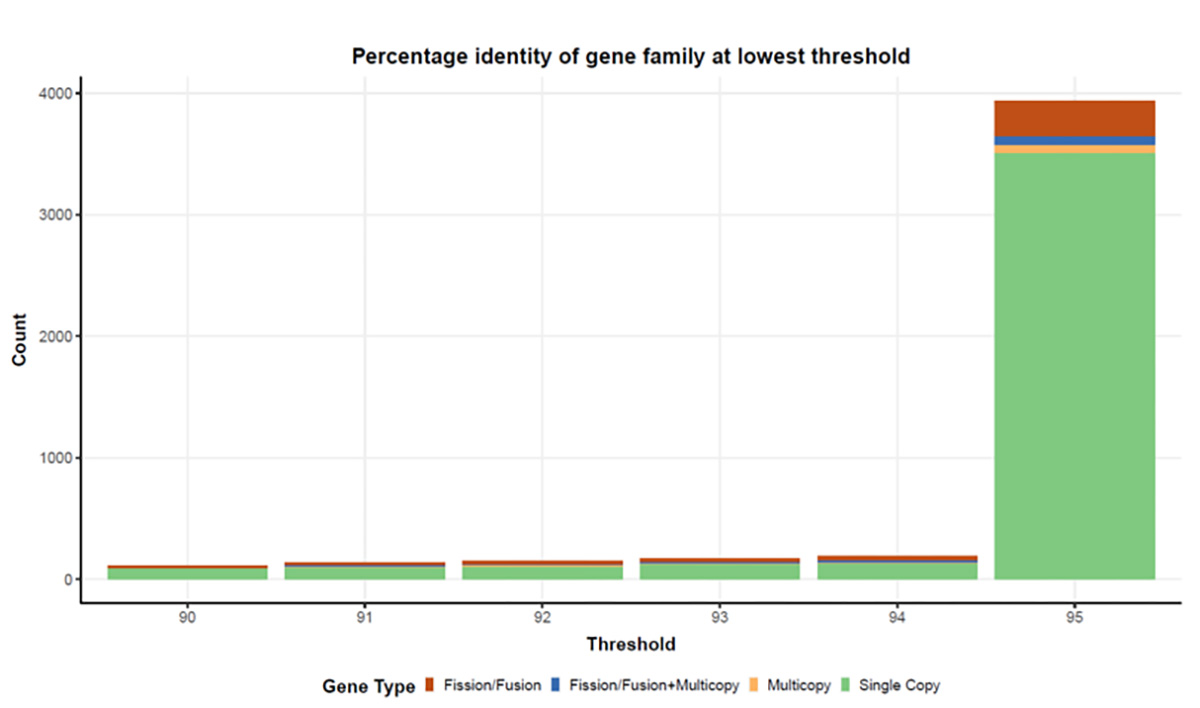

Supplement: Supplementary Figure 1 — Percentage identity of gene family at lowest threshold. Breakdown of percent identity of gene families at the lowest threshold. Identified gene families were classified as either fission/fusion, fission/fusion + multicopy, multicopy, or single copy. [file Image_1.JPEG]
