# Supplementary material for: Whole-Genome Sequencing and Bioinformatic Analysis of Environmental, Agricultural, and Human Campylobacter jejuni Isolates From East Tennessee
Source: Front Microbiol. 2020 Nov 5;11:571064. doi: 10.3389/fmicb.2020.571064 (PMC7674308; doi:10.3389/fmicb.2020.571064)
Supplement: Supplementary Table 1 — Sampling location and date. Sampling source is listed for individual samples collected in this study. Sampling locations are listed by county in Tennessee, along with the collection date for each sample. [file Table_1.docx]

| Sample Name | Sample Source | Sampling Location  (County in Tennessee) | Collection Date |
| --- | --- | --- | --- |
| Human-1 | Human feces | Cocke | 25-Mar-2017 |
| Human-2 | Human feces | Cocke | 31-Mar-2017 |
| Human-3 | Human feces | Washington | 28-Mar-2017 |
| Human-4 | Human feces | Claiborne | 20-Mar-2017 |
| Human-5 | Human feces | Jefferson | 15-Apr-2017 |
| Cow-6 | Cattle feces | Claiborne | 06-Apr-2017 |
| Human-6 | Human feces | Hamblen | 11-Apr-2017 |
| Human-7 | Human feces | Hawkins | 24-Apr-2017 |
| Human-8 | Human feces | Knox | 01-May-2017 |
| Human-9 | Human feces | Sevier | 21-Apr-2017 |
| Human-10 | Human feces | Knox | 26-Apr-2017 |
| Human-11 | Human feces | Grainger | 30-Apr-2017 |
| Human-12 | Human feces | Loudon | 02-May02017 |
| Human-13 | Human feces | Carter | 30-Apr-2017 |
| Human-14 | Human feces | Greene | 04-May-2017 |
| Human-15 | Human feces | Loudon | 09-May-2017 |
| Human-16 | Human feces | Hamblen | 09-May-2017 |
| Human-17 | Human feces | Knox | 09-May-2017 |
| Human-18 | Human feces | Hancock | 09-Jun-2017 |
| Cow-7 | Cattle feces | Claiborne | 06-Apr-2017 |
| Human-19 | Human feces | Greene | 13-Jun-2017 |
| Human-20 | Human feces | Greene | 20-Jun-2017 |
| Human-21 | Human feces | Sullivan | 14-Jun-2017 |
| Human-22 | Human feces | Blount | 27-Jun-2017 |
| Human-23 | Human feces | Blount | 04-Sep-2017 |
| Human-24 | Human feces | Knox | 04-Jul-2017 |
| Human-25 | Human feces | Grainger | 18-Jul-2017 |
| Human-26 | Human feces | Blount | 16-Sep-2017 |
| Human-27 | Human feces | Sevier | 13-Sep-2017 |
| Human-28 | Human feces | Sevier | 11-Oct-2016 |
| Human-29 | Human feces | Knox | 11-Oct-2016 |
| Human-30 | Human feces | Loudon | 29-Jul-2017 |
| Cow-8 | Cattle feces | Claiborne | 06-Apr-2017 |
| Human-31 | Human feces | Loudon | 21-Oct-2016 |
| Human-32 | Human feces | Blount | 20-Oct-2016 |
| Human-33 | Human feces | Sevier | 01-Aug-2017 |
| Human-34 | Human feces | Washington | 26-Oct-2016 |
| Human-35 | Human feces | Loudon | 04-Aug-2017 |
| Human-36 | Human feces | Carter | 25-Oct-2016 |
| Human-37 | Human feces | Sevier | 26-Oct-2016 |
| Human-38 | Human feces | Blount | 26-Oct-2016 |
| Human-39 | Human feces | Monroe | 10-Aug-2017 |
| Human-40 | Human feces | Blount | 04-Aug-2017 |
| Human-41 | Human feces | Knox | 13-Aug-2017 |
| Human-42 | Human feces | Knox | 14-Aug-2017 |
| Human-43 | Human feces | Hawkins | 15-Aug-2017 |
| Human-44 | Human feces | Campbell | 16-Aug-2017 |
| Human-45 | Human feces | Sullivan | 20-Aug-2017 |
| Human-46 | Human feces | Monroe | 29-Sep-2017 |
| Human-47 | Human feces | Knox | 29-Nov-2016 |
| Human-48 | Human feces | Loudon | 30-Aug-2017 |
| Human-49 | Human feces | Knox | 13-Dec-2016 |
| Human-50 | Human feces | Washington | 27-Sep-2017 |
| Human-51 | Human feces | Knox | 10-Sep-2017 |
| Human-52 | Human feces | Washington | 24-Sep-2017 |
| Human-53 | Human feces | Knox | 30-Sep-2017 |
| Human-54 | Human feces | Sullivan | 27-Aug-2017 |
| Human-55 | Human feces | Sullivan | 27-Aug-2017 |
| Chicken (live)-2 | Chicken feces | Knox | 08-May-2017 |
| Chicken (live)-3 | Chicken feces | Knox | 08-May-2017 |
| Chicken (live)-6 | Chicken feces | Knox | 08-May-2017 |
| Non-chicken Bird-1 | Goose feces | Knox | 22-Jun-2017 |
| Non-chicken Bird-2 | Falcon feces | Knox | 22-Sep-2017 |
| Sheep-1 | Sheep feces | Knox | 09-Nov-2016 |
| Sheep-2 | Sheep feces | Knox | 09-Nov-2016 |
| Sheep-3 | Sheep feces | Knox | 09-Nov-2016 |
| Sheep-4 | Sheep feces | Knox | 09-Nov-2016 |
| Water-1 | Holston River | Knox | 07-Dec-2016 |
| Human-56 | Human feces | Hamblen | 20-Feb-2017 |
| Human-57 | Human feces | Carter | 16-Sep-2017 |
| Chicken (live)-8 | Chicken feces | Knox | 17-Apr-2017 |
| Cow-9 | Cattle feces | Grainger | 11-May-2017 |
| Human-58 | Human feces | Sevier | 30-Jan-2017 |
| Human-59 | Human feces | Knox | 07-Feb-2017 |
| Human-60 | Human feces | Jefferson | 01-Feb-2017 |
| Human-61 | Human feces | Grainger | 13-Feb-2017 |
| Human-62 | Human feces | Knox | 21-Feb-2017 |
| Human-63 | Human feces | Knox | 28-Feb-2017 |
| Cow-1 | Cattle feces | Knox | 19-May-2017 |
| Cow-2 | Cattle feces | Knox | 19-May-2017 |
| Cow-3 | Cattle feces | Bledsoe | 12-Jul-2017 |
| Cow-4 | Cattle feces | Sevier | 14-Jul-2017 |
| Cow-5 | Cattle feces | Sevier | 28-Jul-2017 |
| Chicken (live)-1 | Chicken feces | Knox | 08-May-2017 |
| Chicken (live)-4 | Chicken feces | Knox | 08-May-2017 |
| Chicken (live)-5 | Chicken feces | Knox | 08-May-2017 |
| Water-2 | Tennessee River | Knox | 07-Dec-2016 |
